# Effect of psycho-educational interventions on quality of life in patients with implantable cardioverter defibrillators: a meta-analysis of randomized controlled trials

**DOI:** 10.1186/s12955-016-0543-2

**Published:** 2016-09-30

**Authors:** Chi-Wen Kao, Miao-Yi Chen, Ting-Yu Chen, Pai-Hui Lin

**Affiliations:** 1 Department of Nursing, Tri-Service General Hospital, No.325, Sec.2, Chenggong Rd., Taipei, 114 Taiwan; 2 National Defense Medical Center, School of Nursing, No.161, Minchuan E. RD., Sec. 6, Taipei, 114 Taiwan; 3 Department of Nursing, Ching Kuo Institute of Management and Health, No.336, Fu Hsin Rd., Keelung, Taiwan; 4 Chung-Jen College of Nursing, Health Sciences and Management, No.161, Minchuan E. RD., Sec. 6, Taipei, 114 Taiwan

## Abstract

**Background:**

Implantable cardioverter defibrillators (ICD) were developed for primary and secondary prevention of sudden cardiac death. However, ICD recipients’ mortality is significantly predicted by their quality of life (QOL). The aim of this meta-analysis was to evaluate the effects of psycho-educational interventions on QOL in patients with ICDs.

**Methods:**

We systematically searched PubMed, Medline, Cochrane Library, and CINAHL through April 2015 and references of relevant articles. Studies were reviewed if they met following criteria: (1) randomized controlled trial, (2) participants were adults with an ICD, and (3) data were sufficient to evaluate the effect of psychological or educational interventions on QOL measured by the SF-36 or SF-12. Studies were independently selected and their data were extracted by two reviewers. Study quality was evaluated using a modified Jadad scale. The meta-analysis was conducted using the Cochrane Collaboration’s Review Manager Software Package (RevMan 5). Study heterogeneity was assessed by Q statistics and *I*^2^ statistic. Depending on heterogeneity, data were pooled across trials using fixed-effect or random-effect modeling.

**Results:**

Seven randomized controlled trials fulfilled the inclusion and exclusion criteria, and included 1017 participants. The psycho-educational interventions improved physical component summary (PCS) scores in the intervention groups more than in control groups (mean difference 2.08, 95 % CI 0.86 to 3.29, p < 0.001), but did not significantly affect mental component summary (MCS) scores (mean difference 0.84, 95 % CI -1.68 to 3.35, *p* = 0.52).

**Conclusion:**

Our meta-analysis demonstrates that psycho-educational interventions improved the physical component, but not the mental component of QOL in patients with ICDs.

## Background

Implantable cardioverter defibrillator (ICD) has been established as an efficient treatment for primary and secondary prevention of sudden cardiac death [1–4]. Thus, the number of ICD implantations worldwide is growing exponentially every year. In the United States, the ICD implantation rate increased from 6.1 % in 1993 to 46.2 % in 2006, with the average rate significantly increasing 17.9 % per year (95 % confidence interval [CI]: 17.6-18.3 %, *p* < 0.001) [5]. In Australia, the number of ICD implantations nationwide increased from 708 in 2000 to 3198 in 2009, with the implantation rate significantly increasing 19.2 % per year (*p* < 0.001) [6].

Once the device is implanted, patients face various ICD-related changes that affect their quality of life (QOL). For example, patients with ICDs in a systematic review of qualitative studies were found to experience changes that were both physical (e.g., discomfort, sleep disturbance, fatigue) and psychological (e.g., fear of shocks from the ICD, uncertainty about the future, worry about ICD function), which negatively affected their daily activities and QOL [7]. Several other factors related to worse QOL have been identified in patients with ICDs, such as worry about the duration and number of shocks [7–11], uncertainty in daily life [12], anxiety [9, 13, 14], feeling loss of control in life [7, 9], ICD-related concerns [10], lower level of device-related acceptance [13], depression [14, 15], high comorbidity burden [16], Type D personality [17], and negative attitudes toward technology dependency [18]. A significant proportion of ICD recipients experience psychological distress. Indeed, 11–28 % of ICD patients were diagnosed as depressed [19–21], and furthermore a systematic review of 45 studies indicated that 5–41 % of ICD patients had elevated depressive symptoms when assessed by self-report instruments [22]. In the same systematic review with more than 5000 ICD recipients, 11–26 % of patients were diagnosed with anxiety disorders [22]. Thus, healthcare providers should be concerned not only with how long patients live (their quantity of life) with an ICD, but also their QOL.

Drawing overall conclusions about the effect of an ICD on QOL is difficult because of inconsistent results reported [23]. For example, ICD recipients in a systematic review of studies from 1994 to 2006 had better QOL than patients treated with antiarrhythmic medication, but worse than that of the general public or patients with pacemakers [18]. On the other hand, a more recent study found varying results in 80 ICD recipients: 44.7, 20.7, and 34.2 % had no change in QOL, worse QOL, and better QOL, respectively, after implantation [9]. Furthermore, a study conducted in Hong Kong showed that the 85 ICD recipients in this study had a lower level of QOL compared with the general population [13]. In contrast, QOL (SF-36 scores) of 300 patients with ICDs was improved in all domains during the 1-year follow-up, except for the emotional role functioning domain [24].

Quality of life is an important outcome for patients with ICDs since it predicts survival in the first year after implantation [25]. Moreover, the psychological aspects of QOL were cited by the American Heart Association as desirable outcomes of psychological and educational interventions for ICD patients [26]. The return to normal QOL in ICD patients has been shown to be facilitated by structured follow-up and the development of psycho-educational intervention programs [7, 10, 27–29].

During the past decade, various psychological and educational interventions have been designed and provided to ICD patients. Moreover, the effectiveness of these interventions in improving ICD patients’ QOL has been examined in several randomized controlled trials. However, the current evidence for the effectiveness of these trials has not been comprehensively reviewed. Therefore, the aim of this systematic review and meta-analysis was to evaluate the overall effectiveness of psycho-educational interventions on QOL in patients with ICDs.

## Methods

This systematic review and meta-analysis was guided by the Preferred Reporting Items for Systematic Reviews and Meta-Analysis (PRISMA) guideline [30].

### Search strategy

We systematically searched the literature in following databases: Medline, PubMed, Cochrane Library, and CINAHL. The databases were searched from inception to April 2015. Search key words used were “implantable cardioverter defibrillator,” “psychosocial intervention,” “psychological intervention,” “psycho-education intervention,” and “quality of life.” We also used Medical Subject Headings (MeSH) to identify the terminology of key words. To find additional studies and data, we hand searched the references of relevant publications and contacted the authors.

### Study selection criteria

Studies were eligible for review if they met following criteria: (1) a randomized controlled trial, (2) subjects were adult patients with ICDs, (3) a psycho-educational intervention was administered, (4) QOL was measured using the SF-36 or SF-12, and (5) written in English or Chinese.

We selected SF-36 or SF-12 scores as the outcome indicator for QOL to preserve sufficient homogeneity for meta-analysis because these instruments have good properties and are the most widely used to measure QOL in studies on the ICD population internationally [8]. SF-36 or SF-12 scores are calculated and aggregated into two summary scores, a mental component summary score (MCS), and a physical component summary score (PCS) [31, 32].

### Quality assessment

The methodological quality of each trial was independently evaluated by two reviewers (CWK and TYC) using a quality-assessment scale. [33]. This Jadad scale includes 11 items: (1) Was the study designed as randomized? (2) Was the study designed as double blind? (3) Was there a description of withdrawals and drop outs? (4) Were the objects of the study defined? (5) Were the outcome measures defined clearly? (6) Was there a clear description of the inclusion and exclusion criteria? (7) Was the sample size justified? (8) Was there a clear description of the interventions? (9) Was there at least one control group? (10) Was the method used to assess adverse effects described? (11) Were the methods of statistical analysis described? The maximum possible score was 13, and more than nine points was identified as good. Any disagreements in the quality evaluation between two reviewers were resolved by discussion.

### Data extraction

Each selected article was examined to abstract the following data: title of study, authors, publication year, sample size (number of participants in the experimental and control groups), participants’ age and sex, intervention details (type, course, duration, and follow-up), type of control, and the SF-36 or SF-12 score at less than 6 months post-intervention. To assure the accuracy of data extraction, two reviewers abstracted data independently, and discussed any inconsistencies to reach consensus.

### Data analysis and synthesis

The meta-analysis was conducted using RevMan 5.3 (Cochrane Collaboration) software. SF-36 or SF-12 scores are continuous data, which were analyzed as mean differences and 95 % CIs. The statistical heterogeneity among trials was measured by Q statistics and I^2^. Higher values of I^2^ indicate greater heterogeneity, with I^2^ values of 25, 50, and 75 % signifying mild, moderate, and high heterogeneity, respectively [34, 35]. Depending on the heterogeneity findings, data were pooled to estimate the overall effect of all interventions by random-effect or fixed-effect modeling. Publication bias was assessed using the funnel plot method, whose asymmetrical shape shows bias [36].

## Results

### Characteristics of trials

**Fig. 1:**
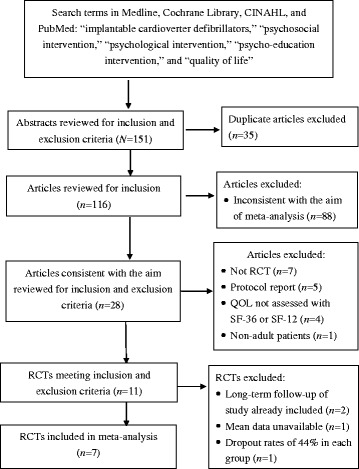
Flow diagram for study selection process

The flow diagram for the study selection process is presented in Fig. 1. Of the 151 articles retrieved from our initial search, we reviewed abstracts and removed 35 duplicate articles and 88 articles that were inconsistent with the aim of the meta-analysis. Among the remaining 28 full-length articles, we excluded 17 studies that did not meet the inclusion criteria. Among the remaining 11 studies, four were not included. One study did not provide the mean and standard deviation of SF-36 scores even after we contacted the authors [37], one study had high dropout rates in each group [38], and two studies [39, 40] analyzed the long-term follow-up outcome of one randomized controlled trial, but we already included the post-intervention outcomes of this trial in our analysis. Therefore, the final data analysis included seven studies [41–47] (Table 1).

**Table 1:** Characteristics of included RCTs

First author, year [ref]	No. of participants (I/C)	Mean age (years)	Mean EF	Intervention	Course	QoL Measure	Measure Time
Berg et al. 2014 [41]	99/97	57.15	32.44 %	Comprehensive cardiac rehabilitation		SF-36	Baseline, 3 m, 6 m, 12 m
				Exercise training; twice a week	12w		
				Psycho-education; once/month for 6 months, and every 2 months for the next 6 months	12 m		
Habibovic et al. 2014 [42]	146/143	55.9	≤ 35 % (63.7 % of patients)	Online problem-solving course based on CBT principles	12w	SF-12	Baseline, 3 m, 6 m, 12 m
Irvine et al. 2011 [43]	94/91	64.39	≤ 35 % (19.7 % of patients)	CBT in eight telephone sessions, psycho-educational book, CD with meditation exercises, muscle relaxation	8w	SF-36	Baseline, 6 m, 12 m
Crössmann et al. 2010 [44]	63/56	60.71	No report	Psychological treatment by phone call	6 m	SF-36	Baseline, 6 m
Kuhl et al. 2009 [45]	15/15	57.44	≤ 35 % (78.2 % of patients)	PACER-CD-ROM-based on psycho-educational CBT	4w	SF-12	Baseline, 1 m
Sears et al. 2007 [46]	15/15	59.77	No report	Components of ICD Shock and Stress Management Program	6w	SF-12	Baseline, 2 m, 4 m
				ICD-specific education			
				Relaxation/stress management training			
				Cognitive-behavioral techniques			
				Group discussion and social support			
Dougherty et al. 2004 [47]	84/84	64.04	33.78 %	Nursing telephone support	8w	SF-12	Baseline, 1 m, 3 m
				ICD recovery management			
				Enhance self-confidence			
				Reduce emotional distress			
				Structure information provided			

*I* intervention, *C* control, *EF* ejection fraction, *QoL* quality of life, *w* weeks, *m* months, *CBT* cognitive-behavioral therapy

These seven studies included 1017 ICD recipients as participants, with 516 randomly assigned to the experimental group, and 501 to the control group. The majority of participants were male (80.4 %, n = 818), with mean age across the studies ranging from 55.9 to 64.4 years, and mean ejection fraction lower than 35 % in five studies [41–43, 45, 47] and not reported in two studies [44, 46]. QOL outcomes were measured in four studies with the SF-12 [42, 45–47], and in three studies using the SF-36 [41, 43, 44].

Table 2 presents the quality assessment of the reviewed studies according to the 11-item Jadad Scale. Five studies had Jadad scores ≥ 9 points, indicating good quality [33]. Two studies had Jadad scores of eight points because they did not meet four of the 11 criteria: double blind, description of dropouts, sample size justified, and report of adverse effects.

**Table 2:** Quality assessment of the included RCTs

References	randomized	double blind	description of drop outs	objects defined	outcome measures defined	Inclusion/ exclusion criteria	sample size justified	description of the interventions	control group	adverse effects	statistical analysis	Jadad Score
Berg et al. [41]	Yes	Yes	Yes	Yes	Yes	Yes	Yes	Yes	Yes	Yes	Yes	13
Irvine et al. [43]	Yes	Yes	Yes	Yes	Yes	Yes	Yes	Yes	Yes	Yes	Yes	13
Habibovic et al. [42]	Yes	Yes	Yes	Yes	Yes	Yes	Yes	Yes	Yes	No	Yes	12
Crössmann et al. [44]	Yes	Yes	Yes	Yes	Yes	Yes	Yes	Yes	Yes	No	Yes	12
Dougherty et al. [47]	Yes	No	Yes	Yes	Yes	Yes	No	Yes	Yes	No	Yes	9
Kuhl et al. [45]	Yes	No	No	Yes	Yes	Yes	No	Yes	Yes	No	Yes	8
Sears et al. [46]	Yes	No	No	Yes	Yes	Yes	No	Yes	Yes	No	Yes	8

### Effect of psycho-educational intervention on QOL

**Fig. 2:**
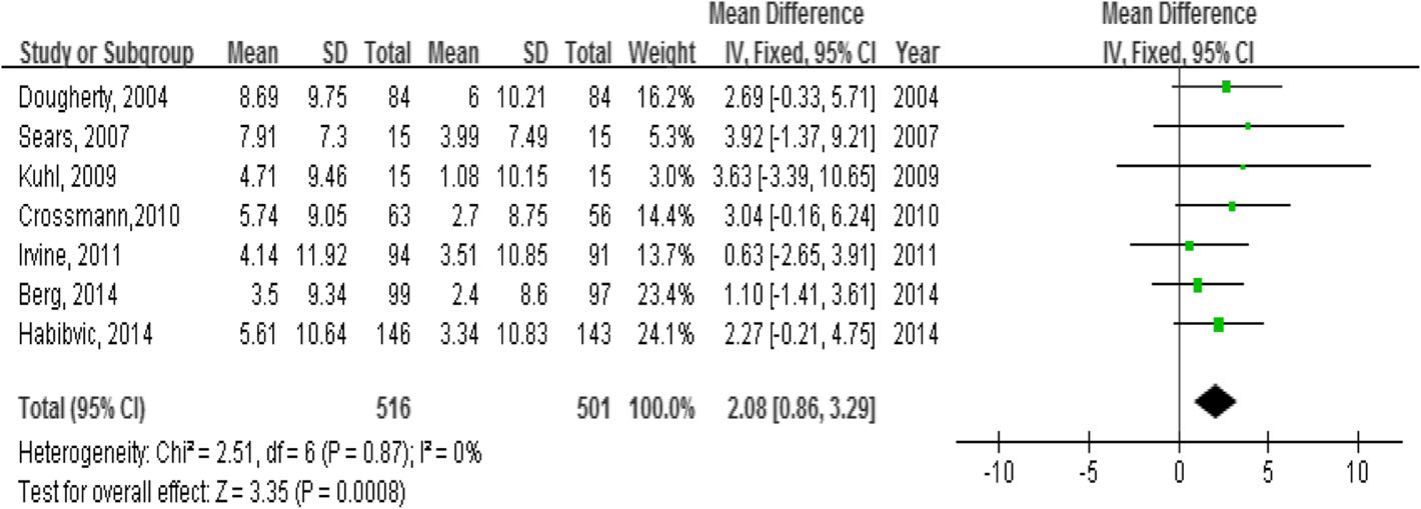
Forest plot of mean differences in physical component summary scores (PCS)

The effect of psycho-educational interventions on QOL was examined by separately considering the effects on PCS and MCS, respectively. For the intervention effect on PCS, we used fixed-effect modeling since no heterogeneity was detected among trials (χ^2^ = 2.51, *p* = 0.87; I^2^ = 0 %). The mean difference in PCS scores between experimental and control groups in all seven studies was estimated as 2.08 (95 % CI 0.86 to 3.29, *p* < 0.001). This result indicates that the psycho-educational interventions significantly improved the physical component of QOL in patients with ICDs (Fig. 2).

**Fig. 3:**
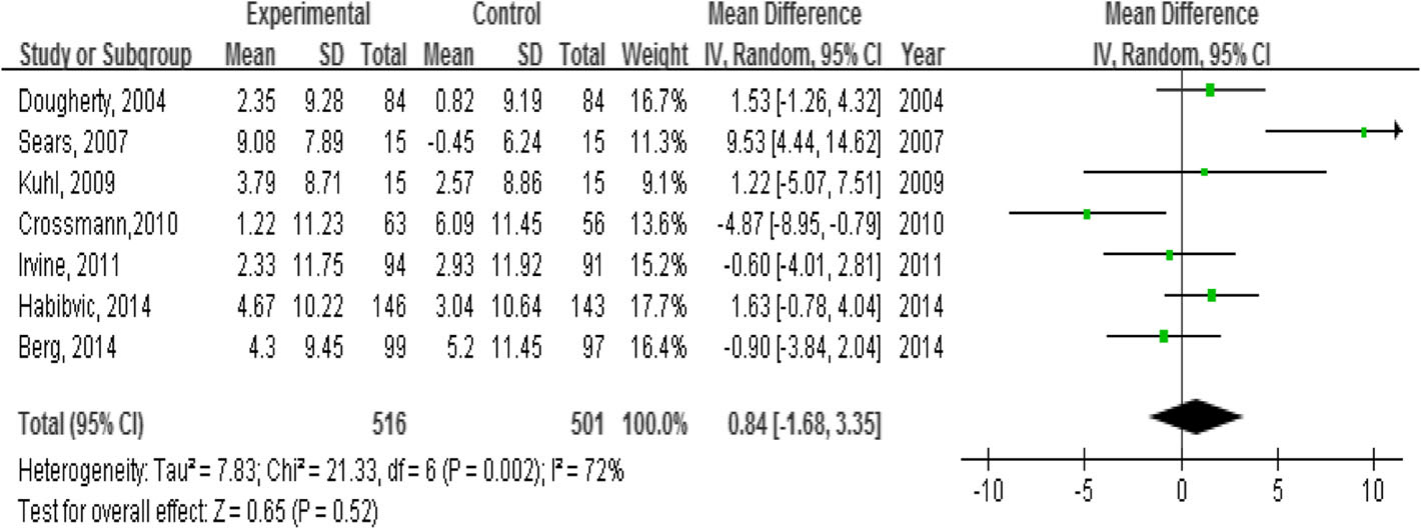
Forest plot of mean differences in mental component summary scores (MCS)

For the intervention effect on MCS, we used random-effect modeling since heterogeneity was identified among trials (χ ^2^ = 21.33, *p* = 0.002; I^2^ = 72 %). The mean difference in MCS scores between the experimental and control groups was estimated as 0.84 (95 % CI -1.68 to 3.35, *p* = 0.52), indicating that the psycho-educational interventions were not effective in improving the mental component of QOL in patients with ICDs (Fig. 3). Since ICD patients’ anxiety and depressive symptoms have been suggested to respond to cognitive behavioral therapy (CBT), we conducted a subgroup analysis of three randomized control trials that used CBT approaches in their interventions [43, 45, 46] to determine if these interventions were effective for the mental component of QOL. The effectiveness of these studies in improving MCS was assessed using random-effect modeling since heterogeneity was identified among trials (χ^2^ = 10.64, *p* = 0.005; I^2^ = 81 %). The mean difference in MCS scores between the experimental and control groups was 3.29 (95 % CI -3.16 to 9.73, *p* = 0.32). The result revealed that using the CBT approach in the psycho-educational interventions for ICD patients was still not significantly effective in improving their mental component of QOL.

As two studies had Jadad scores of 8 points, we eliminated these two studies and conducted a meta-analysis of five randomized controlled trials [41–44, 47]. The psycho-educational interventions still significantly improved the physical component of QOL (95 % CI 0.65 to 3.19, *p* = 0.003), but not the mental component of QOL (95 % CI -3.46 to 1.47, *p* = 0.43) in patients with ICDs.

### Publication bias

Our funnel plot assessment of PCS and MCS showed no evidence of asymmetry, indicating the absence of publication bias.

## Discussion

Our systematic review and meta-analysis of seven randomized controlled trials on 1017 ICD patients provides evidence that psycho-educational interventions effectively improve the physical component but not the mental component of patients’ QOL. Although the majority of recipients regard the ICD as a life-saving medical device that offers them a new chance in life [24, 48], they are affected physically and emotionally by adjustments to the ICD [7]. The psycho-education interventions in the seven reviewed studies were designed to improve recipients’ physical capacity, self-management of ICD-specific care, and self-perceived health [41–47]. The psycho-education interventions included exercise training, psycho-education, problem-solving, ICD-specific education and recovery management, as well as relaxation training. ICD recipients in the experimental groups received more information and strategies to deal with life changes related to ICD implantation, which may have improved their perceived health. Finally, the findings of this meta-analysis indicate that the psycho-educational interventions effectively and significantly improved the physical component of ICD recipients’ QOL (PCS).

Most of the psycho-educational interventions in the seven randomized controlled trials we analyzed aimed to deal with ICD patients’ documented psychological distress and improve their QOL [41–47]. However, our meta-analysis did not detect any effectiveness of these psycho-educational interventions in improving the mental component of QOL. This lack of effect may have been due to the psycho-educational interventions not adequately addressing psychological distress. We also conducted the subgroup analysis of three randomized control trials which used CBT in their interventions [43, 45, 46] to determine whether CBT was effective for the mental component of QOL since ICD patients’ anxiety and depressive symptoms have been suggested to respond to CBT [44, 49]. One study provided CBT in eight telephone consultations with supporting literature [43], another study provided CBT with a patient-assisted computerized intervention program [45], and the third study provided CBT in the context of a 6-week or 1-day ICD shock-and-stress management program [46]. However, the finding cannot support a statistically significant effectiveness.

This lack of effect may have been due to low statistical power of the studies. Among these three studies, two had relatively small samples, with only 15 participants in each group [45, 46]. Another contributing factor may have been limitations of the SF-36, which is a generic measurement to assess health status and QOL. Even though the SF-36 is commonly used in ICD population studies, it may not detect ICD-specific QOL outcomes, especially mental health well-being [16]. Indeed, the mental health subscale of the SF-36 was reported to be less sensitive to changes in mental health than the WHO-Five Well-Being Scale [50]. In addition, MCS scoring assigns positive weights to four domains (vitality, social functioning, role-emotional, and emotional well-being) and negative weights to four other domains (physical functioning, role-physical, bodily pain, and general health). [51]. The ICD patients recruited in these seven studies had poor health due to their heart condition. Most of them had a heart failure history in NYHA class III or IV, and left ventricular ejection fraction < 35 % [41–47]. Therefore, their responses to questions on the negatively weighted last four domains may have driven down their MCS scores.

Among participants in these seven studies, those in five studies experienced ICD shocks [41, 43, 44, 46, 47], those in one study were reported not to experience any ICD shock during the study [45], and the ICD shock experiences of those in another study were not reported [42]. Most people living with an ICD have to cope with stressful or even traumatic events, including having ICD shocks [52]. ICD shocks can be appropriate or inappropriate (not related to therapeutic outcomes), and all ICD shocks adversely affect patients’ mental health [53]. In one qualitative study, ICD patients described their reactions to receiving a shock as losing consciousness, feeling anxious, fearing death and pain, despairing, and worrying about the device not working effectively [37]. Even worse, patients’ psychological distress was greater due to fear of shocks than to actual shocks [54]. In our meta-analysis, we did not detect any effectiveness of psycho-educational interventions on improving the mental component of ICD patients’ QOL (MCS).

### Limitations

The strengths of our study are that it is, to the best of our knowledge, the first meta-analysis of randomized control trials on the effects of psycho-educational interventions on QOL in ICD recipients, the meta-analysis followed the PRISMA guidelines, and we avoided publication bias by hand searching retrieved articles for other studies and tried to contact researchers to ask for unpublished data.

However, this meta-analysis had some limitations. The studies included in the meta-analysis were diverse, with different psycho-educational interventions. To address this issue, we analyzed studies for heterogeneity and, based on these findings, used fixed- or random-effect modeling to pool the data to determine an overall intervention effect. We only included studies published in English or Chinese, which may have introduced publication bias. Finally, only seven studies qualified for inclusion in our meta-analysis, six of which had relatively small samples (<100 participants). Therefore, even though psycho-educational interventions in the original studies [41, 43, 45–47] reported improvements in psychological distress, e.g., depression and anxiety, we did not find that the pooled data from the psycho-educational interventions improved the mental component of QOL (MCS) in ICD recipients.

## Conclusions

The findings of this meta-analysis provide evidence that psycho-educational interventions improved the physical component of QOL (PCS), but not the mental component of QOL (MCS) in patients with ICDs. Nonetheless, we recommend that healthcare providers should still consider the psycho-social adaptation issues of ICD recipients. Identifying effective psycho-educational interventions for improving QOL in ICD recipients will require conducting large-scale randomized clinical trials. Further long-term evaluation follow-up may be needed to examine the durable effectiveness of psycho-educational interventions in patients with ICDs.

## Abbreviations

CBT: Cognitive behavioral therapy
CI: Confidence interval
ICD: Implantable cardioverter defibrillator
MCS: Mental component summary score
MeSH: Medical Subject Heading
NYHA: New York heart association
PCS: Physical component summary score
PRISMA: Preferred Reporting Items for Systematic Reviews and Meta-Analysis
QOL: Quality of life
SF-36: 36-Item Short Form Health Survey
WHO: World Health Organization
